# Effectiveness of a 5-Hydroxytryptamine Type 3 Receptor Antagonist for Treating Nintedanib-Induced Diarrhea: A Prospective Observational Study

**DOI:** 10.3390/jcm14227914

**Published:** 2025-11-07

**Authors:** Toru Arai, Masaki Hirose, Tomoko Kagawa, Naoko Takeuchi, Takehiko Kobayashi, Yoshikazu Inoue, Yoshinobu Matsuda

**Affiliations:** 1 Clinical Research Center, NHO Kinki Chuo Chest Medical Center, Sakai City 591-8555, Osaka, Japan; hirose.masaki.tb@mail.hosp.go.jp (M.H.); kobayashi.takehiko.vz@mail.hosp.go.jp (T.K.); giichiyi@me.com (Y.I.); 2 Department of Respiratory Medicine, NHO Kinki Chuo Chest Medical Center, Sakai City 591-8555, Osaka, Japan; kagawa.tomoko.qw@mail.hosp.go.jp (T.K.); takeuchi.naoko.hb@mail.hosp.go.jp (N.T.); 3 Department of Internal Medicine, Osaka Anti-Tuberculosis Association Osaka Fukujuji Hospital, Neyagawa City 572-0850, Osaka, Japan; 4 Department of Psychosomatic Internal Medicine, NHO Kinki Chuo Chest Medical Center, Sakai City 591-8555, Osaka, Japan; matsuda.yoshinobu.tx@mail.hosp.go.jp

**Keywords:** nintedanib, ramosetron, idiopathic pulmonary fibrosis, progressive pulmonary fibrosis, diarrhea

## Abstract

**Background/Objectives**: Idiopathic pulmonary fibrosis (IPF) is a fibrotic interstitial lung disease (ILD) with a poor prognosis. The prognosis of ILDs showing progressive pulmonary fibrosis (PPF) is poor, similar to that of IPF. Diarrhea is the most frequently observed adverse event in ILDs treated with nintedanib. Managing diarrhea is important for maintaining nintedanib use and improving the prognosis of ILDs. **Methods**: Between October 2022 and March 2025, we enrolled patients with severe nintedanib-induced diarrhea that was uncontrolled by loperamide and/or probiotics. Other drugs were administered to control diarrhea, and the patients were prospectively observed to evaluate stool frequency, stool form score (scores 3, 2, and 1 for watery stool, soft stool without form, and soft stool with form, respectively), quality of life (QOL) using the Japanese version of the irritable bowel syndrome (IBS)-QOL questionnaire, adverse events, and laboratory findings. **Results**: Eleven patients (IPF, *n* = 5; PPF, *n* = 6) were enrolled, and all patients were treated with ramosetron, a 5-hydroxytryptamine type 3 receptor (5-HT_3_) antagonist. Ramosetron was terminated within 3 weeks, before sufficient evaluation, because of insufficient efficacy (*n* = 1) and the discontinuation of nintedanib due to pneumothorax (*n* = 1). Stool frequency and stool form scores decreased significantly after the initiation of ramosetron therapy; however, IBS-QOL did not improve significantly. IBS-QOL correlated with shortness of breath scores but not with stool frequency. No prominent adverse events were associated with ramosetron administration. **Conclusions**: Ramosetron, a 5-HT_3_ antagonist, improved stool frequency and stool form in patients with severe nintedanib-induced diarrhea.

## 1. Introduction

Idiopathic pulmonary fibrosis (IPF) is a fibrotic interstitial lung disease (ILD) of unknown etiology with a poor prognosis [1]. The prognosis of ILDs showing progressive pulmonary fibrosis (PPF) is poor, similar to that of IPF [1]. Nintedanib is a tyrosine kinase inhibitor (TKI) that works by inhibiting signal transduction through the platelet-derived growth factor (PDGF), fibroblast growth factor (FGF), and vascular endothelial growth factor (VEGF) receptors [2], and has been used in the management of patients with IPF and PPF [1,3,4]. It is also used to treat systemic sclerosis-related ILDs [5]. The most common adverse event associated with nintedanib treatment is diarrhea; however, its pathophysiology has not been sufficiently clarified [6]. In the INPULSIS, INBUILD, and SENSCIS trials, more than 60% of patients reported diarrhea [3,4,5]. Anti-diarrheal medications, including loperamide and probiotics, are typically administered. However, a significant proportion of patients continue to experience frequent diarrhea even after treatment. Cottin et al. reported that approximately 30% of patients who experienced diarrhea at least once required a dose reduction in or the discontinuation of nintedanib in the INBUILD trial [7]. Hence, new treatments for nintedanib-induced diarrhea are required to improve the survival and quality of life (QOL) of these patients.

Ramosetron, a 5-hydroxytryptamine (5-HT) type 3 (5-HT_3_) receptor antagonist used to treat diarrhea-predominant irritable bowel syndrome (IBS) [8], may be a candidate drug for treating nintedanib-induced diarrhea [9]. We previously reported on two patients with nintedanib-induced diarrhea who were successfully treated with ramosetron [9]. Hence, we prospectively enrolled patients with severe nintedanib-induced diarrhea unresponsive to loperamide and observed the symptoms and QOL of the patients after commencing ramosetron.

## 2. Materials and Methods

This prospective observational study was approved by the Institutional Review Board of the NHO Kinki Chuo Chest Medical Center (KCCMC; approval number: Rin2022-072; 20 October 2022). Written informed consent was obtained from all participants.

From October 2022 to March 2025, we enrolled patients with severe nintedanib-induced diarrhea who satisfied the following criteria: (A) diarrhea was evaluated as grade 3 according to the Common Terminology Criteria for Adverse Events (CTCAE) [10], (B) diarrhea was subjectively intolerable under treatment with 1–2 mg daily of loperamide, or (C) medication other than loperamide was needed to control diarrhea according to the decision of the chief managing doctor. If drugs other than loperamide and probiotics were administered to control diarrhea and nintedanib was continued, the patients were prospectively observed to evaluate stool frequency, stool type, and other subjective symptoms, as well as laboratory findings. QOL was also evaluated. Ramosetron was the only drug added to nintedanib. The underlying ILDs in the patients were IPF or non-IPF with PPF.

### 2.1. Diagnosis of Underlying ILD

IPF was diagnosed based on the international guidelines updated in 2022 [1]. Patients with non-IPF were evaluated to determine whether they satisfied the criteria for PPF according to international guidelines [1]. The diagnosis of sarcoidosis was confirmed based on the Japan Society of Sarcoidosis and Other Granulomatous Disorders 2015 diagnostic criteria [11]. Sjögren syndrome [12], systemic sclerosis (SSc) [13], and pulmonary alveolar proteinosis (PAP) [14] were diagnosed using conventional diagnostic criteria.

### 2.2. Treatment and Evaluation Schedule

**Figure 1:**
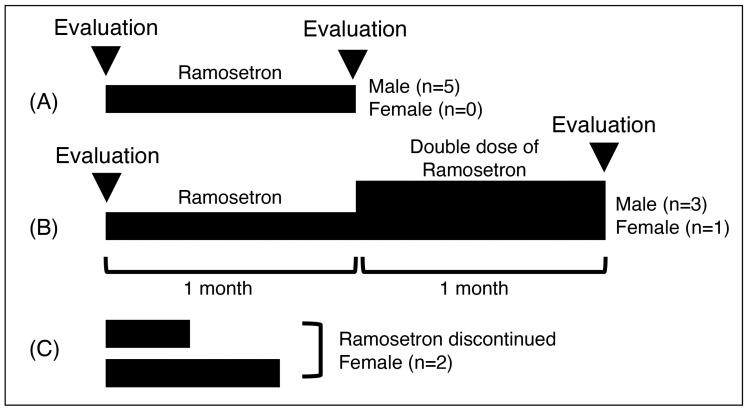
The standard daily dose of ramosetron was 5 mg for men and 2.5 mg for women. If the efficacy of ramosetron was insufficient, its dose could be doubled 1 month after commencing the standard dose. Before and after the 1-month treatment with standard ramosetron (**A**) and double-dose ramosetron (**B**), daily stool frequency, stool form score, loperamide dose, and irritable bowel syndrome quality of life were evaluated (arrowheads). Two patients discontinued ramosetron therapy, and the efficacy of ramosetron could not be evaluated (**C**).

The treatment and evaluation schedules are shown in Figure 1. The standard doses of ramosetron for IBS are 5 and 2.5 mg daily for males and females, respectively [15]. Ramosetron was initially administered at the standard dose. Based on each patient’s symptoms, the dose of ramosetron was doubled after 1 month, if necessary. The loperamide dose could be changed according to the severity of diarrhea. The final evaluation was performed after 1 month of treatment with a standard or double dose of ramosetron. Diarrhea, QOL, loperamide dose, and other laboratory findings were evaluated at the start of ramosetron and 1 month after the start of the final dose of ramosetron.

### 2.3. Questionnaire

QOL associated with abdominal symptoms was evaluated using the IBS-QOL questionnaire devised by Patrick et al. [16], translated into Japanese and validated by Kanazawa et al. [17]. The higher the IBS-QOL score, the better the QOL. This questionnaire was devised for patients with IBS and not specifically for diarrhea. However, because nintedanib-induced diarrhea affects the physical, mental, and social aspects of QOL, the questionnaire was considered suitable for these patients. The subclass parameters of IBS-QOL, including “dysphoria,” “interference with activity,” “body image,” “health worry,” “food avoidance,” “social reaction,” “sexual,” and “relationships,” were also evaluated [18]. The minimal clinically important difference (MCID) in the IBS-QOL is 10 points [19]; this MCID was adopted to evaluate the QOL of patients with nintedanib-induced diarrhea.

### 2.4. Evaluation of Nintedanib-Induced Diarrhea

The frequency (stools per day), stool form, and the dose of loperamide needed to improve diarrhea were evaluated 1 month after the final dose of ramosetron. Stool form was classified into watery stool, soft stool with no form, and soft stool with form, as described in the IBS guidelines [20], scored as 3, 2, or 1, respectively. We hypothesized that the severity of diarrhea could be evaluated using stool frequency and stool form; hence, we calculated stools per day multiplied by stool form scores and termed it the diarrhea severity score.

### 2.5. Clinical Findings at the Commencement of Ramosetron

All clinical evaluations, except the IBS-QOL questionnaire, were performed as part of general practice for patients with nintedanib-treated ILD. Clinical findings, including age, sex, body mass index (BMI), smoking status, mMRC score [21], pulmonary function test results, and laboratory test results at the time of ramosetron commencement, were obtained retrospectively from the medical records. Pulmonary function tests, including forced vital capacity (FVC) and diffusing capacity of carbon monoxide (DLco), were performed using a Chestac 8080 spirometer (Chest, Tokyo, Japan). ILD biomarkers, including serum levels of Krebs von den Lungen-6 (KL-6) and surfactant protein (SP)-D [22], were retrieved from medical records.

### 2.6. Safety

The adverse events associated with new drugs used for the management of severe nintedanib-induced diarrhea, in addition to loperamide and probiotics, were evaluated.

### 2.7. Statistical Analysis

Continuous variables are presented as medians and ranges, and categorical variables are presented as numbers.

The Wilcoxon signed-rank test was used to compare non-parametric distributions of paired observations for continuous variables. Categorical variables were compared using Fisher’s exact test. Correlations between two parameters were examined using Spearman’s rank correlation test. All statistical analyses were performed using SPSS for Macintosh version 29 (IBM Corp., Armonk, NY, USA). Statistical significance was set at *p* < 0.05.

## 3. Results

### 3.1. Patient Demographics

Eleven patients were enrolled in the study. The patient demographics at baseline are presented in Table 1. The underlying ILDs included IPF (*n* = 5), pulmonary fibrosis related to autoimmune PAP (*n* = 3), Sjögren syndrome-related ILD (*n* = 1), systemic sclerosis-related ILD (*n* = 1), and sarcoidosis (*n* = 1). The median age was 66 years, and 8 patients were men. The median %FVC (range) was 62.8 (39.0–146.2), and the median BMI was 22.6 (14.6–29.5). The daily doses of nintedanib were 300 mg (*n* = 5) and 200 mg (*n* = 6). One patient was administered a proton pump inhibitor; however, other potential causative drugs for diarrhea were not administered.

### 3.2. Ramosetron Treatment

Two patients stopped ramosetron within 3 weeks and could not be evaluated in detail. No significant effects were observed within 10 days in one patient, and in the other, the diarrhea settled because of the discontinuation of nintedanib due to pneumothorax, which occurred 20 days after ramosetron commencement. After the subjective evaluation of each patient, the dose of ramosetron was doubled to 10 mg daily in three men and to 5 mg daily in one woman because of insufficient effects (*n* = 2) or the expectation of greater effects (*n* = 2).

### 3.3. Severity of Diarrhea, Including Frequency and Stool Form, Before and After Commencing Ramosetron

We examined the severity of diarrhea in nine patients who were taking the final dose of ramosetron for 1 month. Before commencing ramosetron, the median daily stool frequency was 6.0 (4.0–7.0), and the median stool form score was 2 (2–3), corresponding to a soft stool without form. The median loperamide dose was 2 (1–3) mg daily. One month after commencing treatment with the final dose of ramosetron, the median daily stool frequency was 4.0 (2.0–5.7), and the median stool form score was 1 (1–2). The frequency and stool form scores significantly improved (*p* = 0.011 for both, Mann–Whitney U test; Table 2). The diarrhea severity score also significantly improved. The loperamide dose did not change significantly: it decreased (*n* = 3), remained similar (*n* = 5), or increased (*n* = 1). Improvement in the CTCAE grade of diarrhea and stool form score ≥1 was observed in six and seven of the nine patients (66.7% and 77.7%), respectively.

### 3.4. IBS-QOL Evaluation

IBS-QOL was evaluated in eight of the nine diarrhea-evaluable patients (Table 2); one patient could not sufficiently answer the IBS-QOL questionnaire. The total IBS-QOL scores increased in six of the eight patients after commencing ramosetron; however, the improvement was not significant (*p* = 0.161, Mann–Whitney U test), and it exceeded the MCID (10 points) in only two patients (25%).

The IBS-QOL subclasses were also compared before and after commencing ramosetron. There were no significant differences in any subclass scores (Table 3). Correlations between the total and subclass scores of the IBS-QOL and parameters of ILD severity (mMRC) and diarrhea severity (stools per day) were examined (Table 4). The mMRC score was significantly correlated with the total score and the “dysphoria” and “interference with activity” subclass scores (*p* = 0.001, *p* = 0.019, and *p* = 0.012, respectively; Spearman’s rank correlation). Stools per day were significantly correlated with the “dysphoria” and “food avoidance” subclasses (*p* = 0.022 and *p* = 0.030, respectively; Spearman’s rank correlation).

### 3.5. Final Observation

Nine patients in whom the effects of ramosetron on diarrhea could be evaluated continued taking nintedanib while managing their diarrhea with ramosetron at the final observation. The median ramosetron intake duration was 217 days (60–861; Table 2). One patient who stopped ramosetron within 10 days because of insufficient effects was able to take nintedanib with re-administration of ramosetron 2 months after the initial termination, achieving good control of diarrhea. Another patient who stopped ramosetron treatment because nintedanib was discontinued due to pneumothorax survived 841 days after the first intake of ramosetron.

In seven out of the nine patients who could continue taking nintedanib under the management with ramosetron, we could have compared the %FVC before ramosetron initiation and at the final observation. Median absolute decline (range) in %FVC per one year between ramosetron initiation to final observation was 1.90% (−0.10–6.57) and absolute decline %FVC ≧ 5%, which is one of the parameters of PPF (1), was observed in only one patient. The other two patients could not undergo a pulmonary function test at the final observation because of a giant cyst in one patient and severe restrictive ventilation in another patient.

### 3.6. Safety of Ramosetron

Adverse events associated with ramosetron administration, including constipation, hard stool, liver dysfunction, and a decrease in blood cell counts, which were reported according to the clinical trial [8], were not observed in this study.

## 4. Discussion

This is the first prospective observational study to demonstrate the effects of anti-diarrheal drugs other than conventional drugs like loperamide on nintedanib-induced diarrhea. Ramosetron was used as an additional treatment for diarrhea. The decision to commence ramosetron was made by a pulmonologist with the informed consent of each patient with ILD, and our study facilitated the evaluation of its effectiveness. The frequency of diarrhea and stool form scores significantly improved after the initiation of ramosetron therapy; however, we could not clearly show an improvement in QOL evaluated by the IBS-QOL questionnaire. In addition, the management of diarrhea by ramosetron might have extended the duration of nintedanib treatment and led to the maintenance of %FVC.

The mechanism of nintedanib-induced diarrhea has not yet been clarified; however, intestinal mucosal damage caused by the inhibition of TKIs of PDGF, FGF, and VEGF receptors by nintedanib [2] is thought to be associated with its occurrence [6]. In addition, the serotonin pathway may be involved in nintedanib-induced diarrhea [9]. Serotonin (5-HT) is synthesized from tryptophan by tryptophan hydroxylase (TpH) in the endocrine (EC) cells of the gut epithelium [23]. Under normal conditions, most of the secreted 5-HT is transported through the 5-HT reuptake transporter (SERT) to enterocytes for degradation or to the platelets for storage [23,24,25]. During colitis and diarrhea, the number of EC cells often increases, resulting in higher levels of secreted 5-HT [23,26], while the expression of SERT is reduced. The elevated mucosal 5-HT induced by these mechanisms stimulates the 5-HT_3_ receptor located on vagal sensory fibers and induces the release of acetylcholine, subsequently leading to smooth muscle contraction and diarrhea [23,27]. SERT gene polymorphisms are associated with the occurrence of diarrhea induced by imatinib, another TKI [28]. Bosutinib is one of the most potent diarrhea-inducing TKIs; it inhibits SERT activity by 71% due to its off-target effects [29]. Nintedanib may similarly inhibit SERT and upregulate the local concentration of 5-HT in the intestinal mucosa. As a result, the increased 5-HT enhances intestinal movement through 5-HT_3_ receptors located on the intestinal mucosa, resulting in diarrhea.

Ramosetron, a 5-HT_3_ receptor antagonist, is used to treat diarrhea in IBS [8,15,19]. Similarly, it can reduce nintedanib-induced diarrhea. Herein, stool frequency and stool form improved significantly. Improvements in the CTCAE grade of diarrhea and stool form scores were observed in 66.7% and 77.7% of patients, respectively. We previously reported on two patients with ILD whose nintedanib-induced diarrhea was controlled using ramosetron [9]. In these patients, diarrhea was dramatically reduced by ramosetron administration within 3 days, and the necessary dose of loperamide could also be reduced. Compared with our previous report, the results of the present study may be less favorable, and the dose of loperamide could not be reduced in more than half of the patients; however, the 1-month response rate of ramosetron in patients with nintedanib-induced diarrhea might be better than that in patients with IBS (35.36%) [8]. The difference in the response rate to ramosetron might be due to differences in the contributions of the 5-HT pathway to nintedanib-induced diarrhea and diarrhea secondary to IBS. IBS is believed to be caused by several physical, mental, and social factors [20].

We demonstrated the significant efficacy of ramosetron on stool frequency per day and stool form scores; however, we could not show an improvement in the IBS-QOL score before and after commencing ramosetron. According to our analysis, the total IBS-QOL score and the “interference with activity” subclass score were more closely associated with mMRC, one of the most important parameters associated with QOL in IPF [30], than daily stool frequency, while “avoidance of food” was associated with daily stool frequency. We believe that we could not find any difference in IBS-QOL before and after commencing ramosetron in this study because the QOL of patients with ILD treated with nintedanib was determined by both ILD and diarrhea parameters, unlike that in patients with IBS. We will adopt daily stool frequency or stool form score, instead of the IBS-QOL score, as the primary endpoint in future randomized clinical trials evaluating the anti-diarrheal effects of ramosetron on nintedanib-induced diarrhea.

All of 9 patients, in whom effectiveness of ramosetorn for nintedanib-induced diarrhea were evaluated, could keep taking nintedanib under the management of diarrhea with ramosetron until the final observation. The median duration from the initiation of nintedanib treatment to the final observation was 1020 days (482–2374). Based on the 1- year discontinuation rate of nintedanib, 50%, in the real-world patients [31], management with ramosetron might have been associated with long term continuation of nintedanib therapy. Recently, Satonaga et al. reported that management with ramosetron prolonged the duration of the nintedanib therapy compared with that without ramosetron based on their retrospective study [32]. Ramosetron administration was a significant predictor of nintedanib continuation after the adjustment with age, BMI, and %FVC.

Clinical trials evaluating the efficacy of ramosetron against IBS revealed adverse events such as constipation and hard stools, and the frequency of these events in the ramosetron arm was higher than that in the placebo arm [19]; however, we did not observe such adverse events in this study. Hence, ramosetron may be safe to use in patients with severe nintedanib-induced diarrhea, although loperamide dose adjustment might be needed [19]. In mild-to-moderate nintedanib-induced diarrhea, gastrointestinal adverse events, including constipation and hard stools, must be cautiously observed.

This study has some limitations. First, it was performed at a single institute, and only a small number of patients were included. Second, QOL could not be evaluated for all participants. Third, two patients stopped taking nintedanib, and one of them stopped ramosetron because of insufficient anti-diarrheal effects; however, we believe it is best not to evaluate the effects of ramosetron when the intake period is only ten days. Fourth, the efficacy and safety of ramosetron for milder nintedanib-induced diarrhea without loperamide remain unknown, and the use of ramosetron as a first-line treatment is controversial. Fifth, ramosetron is approved only in Japan and some Asian countries [33], and it is not available in USA and European countries. Hence, generalizability of our study about ramosetron is an important problem. Other 5-HT_3_ receptor antagonists, alosetron [20] and ondansetron [34], etc., might be potential drugs for nintedanib-induced diarrhea. Alosetron is now approved for limited population of IBS only in USA due to the concerns about possibility of ischemic colitis [35,36]. Ondansetron is used as an antiemetic drug for the treatment of malignant diseases [34] for short duration usually≦5 days widely in the world. Nintedanib is used for non-small cell lung cancer combined with docetaxel [37] and ondansetron could be used with this combination therapy; however, no reports have focused on the anti-diarrheal effects of ondansetron. It is supposed to be effective for diarrhea-predominant IBS from the results of clinical trial [38,39]. However, it is not approved for IBS [33]. Moreover, efficacy of these drugs for nintedanib-induced diarrhea has not been reported. Hence, future clinical trials are needed to improve the management of nintedanib-induced diarrhea.

## 5. Conclusions

Ramosetron, a 5-HT_3_ receptor antagonist, may be useful for managing nintedanib-induced diarrhea, and its efficacy deserves to be clarified in future randomized controlled trials.

## Figures and Tables

**Table 1:** Patient demographics.

Parameters	Frequency or Median (Range)
**<Baseline>**	
Gender, Male/Female	8/3
Age, yrs	74 (62–84)
Smoking. NS/ES or CS	4/7
Diagnosis of ILDs, IPF/non-IPF *	5/6
BMI, kg/m^2^	22.6 (14.6–29.5)
mMRC, <2/2≤	3/8
%FVC, %	62.8 (39.0–146.2)
%DLco, %	43.1 (29.8–63.6)
KL-6, x100 U/mL	8.50 (5.87–12.02)
SP-D, ng/mL	287.4 (120.4–801.5)
Loperamide, yes/no	11/0
Probiotics, yes/no	9/2
Proton pump inhibitor, yes/no	1/10
Corticosteroids, yes/no	1/10
Immunosuppressants, yes/no	0/11
Dose of nintedanib, 300/200 (mg/day)	5/6
Days from nintedanib to onset of diarrhea	143 (1–359)
**<After Ramosetron initiation>**	
Days from diarrhea to ramosetron	489 (105–1211)
Nintedanib usage at the final observation	10/1 **
Initial dose of ramosetron	
For male, 5 mg/10 mg	8/0
For female, 2.5 mg/5 mg	3/0
Final dose of ramosetron	
For male, 5 mg/10 mg	5/3
For female, 2.5 mg/5 mg	0 ^#^/1
Ramosetron continuation at the final observation, yes/no	9/2 ^#^
Ramosetron continuation period ^†^, days	200 (10–861)
Outcome ^†^, alive/dead	11/0
Days from ramosetron to final observation ^‡^	217 (60–861)
Days from nintedanib initiation to final observation ^‡^	1020 (482–2374)

Abbreviations: BMI, body mass index; CS, current smoker; ES, ex-smoker; ILDs, interstitial lung diseases; IPF, idiopathic pulmonary fibrosis; mMRC, modified Medical Research Council score for shortness of breath; NS, non-smoker; %FVC, percent predicted value of forced vital capacity; %DLco, percent predicted value of diffusing capacity of carbon monoxide; SP-D, surfactant protein-D. *: Non-IPF included sarcoidosis (*n* = 1), systemic sclerosis-related ILD (*n* = 1), Sjögren syndrome-related ILD (*n* = 1), and autoimmune pulmonary alveolar proteinosis-related pulmonary fibrosis (*n* = 3). **: One patient stopped nintedanib due to the incidence of pneumothorax. ^#^: Two female patients stopped ramosetron because of nintedanib discontinuation (*n* = 1, 20 days after initiation) due to pneumothorax and insufficient effect of ramosetron (*n* = 1, 10 days after initiation). The latter patient re-started, and diarrhea was subjectively improved. ^†^: for all patients, ^‡^: for patients who continued ramosetron from the initiation to final observation (*n* = 9).

**Table 2:** Status, treatment, and QOL of diarrhea before and after the start of ramosetron (*n* = 9 *).

	Before Ramosetron	After Ramosetron ^¶,†^	*p*-Value
Dose of ramosetron			
For males, 5 mg/10 mg	8/0	5/3 ^¶^	NE
For females, 2.5 mg/5 mg	1/0	0/1 ^¶^	NE
Stool per day	6.0 (4–7)	4.0 (2.0–5.7)	0.011
Diarrhea CTCAE, 1/2/3	0/7/2	4/5/0	0.014
Downgrade of CTCAE, yes/no	NA	6/3	NA
Stool form scores ^#^	2 (2–3)	1 (1–2)	0.011
Downgrade of stool form, yes/no	NA	7/2	NA
Diarrhea severity score ^‡^	12 (8–21)	4 (2–10)	0.008
Loperamide dose, mg/day	2 (1–3)	2 (0–3)	0.257
Loperamide dose, decrease/similar/increase	NA	3/5/1	NA
IBS QOL **	66.5 (47.1–82.4)	73.2 (49.3–89)	0.161
Improvement of IBS QOL, yes/no	NA	6/2	NA
Improvement of IBS QOL > MCID ^§^, yes/no	NA	2/6	NA

Abbreviations: IBS, irritable bowel syndrome; QOL, quality of life; MCID, minimal clinically important difference; NA, not available; NE, not evaluated. *: Wilcoxon signed-rank test was used to compare the non-parametric distribution of the paired observations. Two patients stopped ramosetron within 3 weeks after initiation and were excluded from this analysis. **: IBS-QOL could not be evaluated in one patient, and IBS-QOL before and after the start of ramosetron was compared in eight patients. ^#^: Stool form was classified into watery (score 3), soft stool without form (score 2), soft stool with form (score 1), and normal stool (score 0). ^¶^: Dose of ramosetron was doubled to 10 mg daily in the three male patients and to 5 mg daily in one female patient. ^§^: MCID for IBS-QOL is 10. ^†^: Median duration of ramosetron intake (range) until final observation was 217 days (60–861 days) (* *n* = 9). All nine patients continued ramosetron. ‡: Diarrhea severity score was calculated as stools per day multiplied by stool form score.

**Table 3:** IBS QOL; scales for subclasses (*n* = 8).

	Before Ramosetron	After Ramosetron ^¶^	*p*-Value
Dysphoria	59.4 (18.8–81.3)	60.9 (31.3–87.5)	0.553
Interference with activity	57.1 (21.4–67.9)	58.9 (21.4–82.1)	0.345
Body Image	84.3 (50–100)	87.5 (50–100)	0.450
Health worry	66.7 (66.7–91.7)	83.3 (66.7–91.7)	0.058
Food avoidance	58.3 (41.7–100)	75 (41.7–100)	0.236
Social reaction	87.5 (56.3–100)	84.4 (62.5–100)	0.751
Sexual	100 (0–100)	100 (0–100)	0.593
Relationships	66.7 (25.0–91.7)	83.3 (58.3–100)	0.106

Abbreviations: IBS, irritable bowel syndrome; QOL, quality of life. Wilcoxon signed-rank test was used to compare the non-parametric distribution of paired observations for the continuous variables. ^¶^: Dose of ramosetron was doubled to 10 mg daily in three male patients and to 5 mg daily in one female patient.

**Table 4:** Correlation of severity parameters of ILDs and stool parameters with IBS-QOL (total scores and scores for subclasses) (Spearman’s rank correlation).

	mMRC		Stool Per Day	
	Rho	*p*-Value	Rho	*p*-Value
Total score	−0.914	0.001	−0.586	0.127
Dysphoria	−0.791	0.019	−0.781	0.022
Interference with activity	−0.823	0.012	−0.450	0.263
Body Image	−0.398	0.329	−0.098	0.817
Health worry	−0.542	0.165	0.141	0.739
Food avoidance	−0.567	0.143	−0.755	0.030
Social reaction	−0.456	0.256	−0.200	0.635
Sexual	−0.156	0.713	−0.391	0.338
Relationships	−0.889	0.003	−0.319	0.441

Abbreviations: IBS, irritable bowel syndrome; ILDs, interstitial lung diseases; mMRC, modified Medical Research Council score for shortness of breath; QOL, quality of life. Association between two parameters was examined using Spearman’s rank correlation.

## Data Availability

The data presented in this study are available on request from the corresponding author; however, this access may require additional ethical approval. Requests may also be declined if they conflict with our future research plans.

## Approval of IBS-QOL Questionnaire Use

The Japanese version of the IBS-QOL questionnaire was used with the approval of Motoyori Kanazawa and Shin Fukudo (Department of Behavioral Medicine, Tohoku University Graduate School of Medicine).

## Abbreviations

The following abbreviations are used in this manuscript:

IPF	Idiopathic Pulmonary Fibrosis
ILD	Interstitial Lung Disease
PPF	Progressive Pulmonary Fibrosis
TKI	Tyrosine Kinase Inhibitor
PDGF	Platelet-Derived Growth Factor
FGF	Fibroblast Growth Factor
VEGF	Vascular Endothelial Growth Factor
QOL	Quality of Life
IBS	Irritable Bowel Syndrome
5-HT	5-Hydroxytryptamine (Serotonin)
5-HT3	5-Hydroxytryptamine Type 3 Receptor
NHO KCCMC	National Hospital Organization Kinki Chuo Chest Medical Center
CTCAE	Common Terminology Criteria for Adverse Events
MCID	Minimal Clinically Important Difference
mMRC	Modified Medical Research Council Dyspnea Scale
FVC	Forced Vital Capacity
DLco	Diffusing Capacity of the Lung for Carbon Monoxide
KL-6	Krebs von den Lungen-6
SP-D	Surfactant Protein-D
PAP	Pulmonary Alveolar Proteinosis
SSc	Systemic Sclerosis
SERT	Serotonin Transporter
TpH	Tryptophan Hydroxylase
EC Cells	Enterochromaffin Cells
SPSS	Statistical Package for the Social Sciences
IBS-QOL	Irritable Bowel Syndrome–Quality of Life Questionnaire
